# Preoperative consultation for determining the appropriate transfusion strategy

**DOI:** 10.1007/s44313-024-00021-x

**Published:** 2024-06-07

**Authors:** Ka-Won Kang

**Affiliations:** Department of Internal Medicine, Division of Hematology-Oncology, Korea University College of Medicine, 73, Goryeodae-ro, Seongbuk-gu, Seoul, 02841 the Republic of Korea

**Keywords:** Preoperative care, Anemia, Thrombocytopenia, Blood transfusion, Patient blood management

## Abstract

Surgical patients are at risk of postoperative complications and mortality, necessitating preoperative patient optimization through the identification and correction of modifiable risk factors. Although preoperative platelet transfusions aim to reduce the risk of bleeding, their efficacy remains uncertain. Similarly, red blood cell transfusion in patients with anemia does not reduce the risk of postoperative mortality and may exacerbate complications. Therefore, developing individualized strategies that focus on correcting preoperative complete blood count abnormalities and minimizing transfusion requirements are essential. This review aimed to examine complete blood count abnormalities and appropriate transfusion strategies to minimize postoperative complications.

## Introduction

Approximately 30% of all surgical patients experience postoperative complications, with 3% resulting in mortality [1, 2]. The initial strategy to mitigate these risks involves identifying and actively addressing remediable or modifiable risk factors before surgery through preoperative patient optimization [3]. This review aimed to discuss complete blood count (CBC) abnormalities, with emphasis on optimizing transfusion practices to minimize the incidence of postoperative complications.

### Preoperative screening tests for hemostatic defects

Prior to evaluating CBC abnormalities and determining appropriate transfusion strategies, hemostatic defects such as inherited coagulation and platelet (PLT) disorders must be ruled out. In major studies concerning transfusion based on abnormal CBC findings, patients with inherited hemostatic defects were either excluded or not further analyzed. Therefore, these patients should be categorized separately and undergo preoperative patient optimization according to the specific guidelines for each condition.

Identifying these patients is crucial. Excessive postoperative bleeding, as reported by surgeons, occurs in approximately 3% of all surgical procedures [4]. Laboratory assessments including, PLT count, prothrombin time, and activated partial thromboplastin time (aPTT) tests, have been employed to predict the risk of excessive postoperative bleeding. However, these preoperative screening tests exhibit no correlation with surgery-induced hemorrhage, exhibiting low sensitivity (<35%) and a positive predictive value (<3%) [5, 6]. Furthermore, given the lack of evidence supporting the efficacy of such tests in reducing postoperative bleeding, the routine implementation of these conventional screenings across all surgical patients is not warranted [6, 7].

Preoperative screening tests for hemostatic defects are deemed insufficient for several reasons. First, most patients with inherited coagulation and PLT disorders are diagnosed early in life, typically before the age of 10 years [8–12]. For adult patients undergoing surgery, obtaining a patient history is a faster and more cost-effective method for detecting inherited hemostatic defects. Second, the predominant cause of postoperative bleeding is not hemostatic failure but rather surgical or technical complications, which can be appropriately addressed by surgical interventions [5, 13]. Lastly, the traditional tests (PLT count, prothrombin time, and aPTT tests) are primarily designed to detect static disorders. Consequently, preoperative tests cannot reliably predict hemostatic disorders that may arise during surgery, such as disseminated intravascular coagulation, hyperfibrinolysis, and thrombocytopenia. 

In conclusion, the risk of bleeding associated with surgery should be initially assessed through thorough history taking and physical examination, followed by selective preoperative screening tests in individuals deemed at high risk. Table 1 summarizes the relevant questions for this purpose, while Table 2 outlines the specific preoperative screening tests for each bleeding risk [14].

**Table 1:** Appropriate inquiry for screening possible abnormal hemostasis

• Has there been any history of bleeding disorders in your family, such as excessive bleeding tendencies or abnormal bleeding incidents?
• Have you experienced any episodes of prolonged bleeding following surgical procedures or childbirth?
• In the past, have healthcare professionals needed to revisit a surgical site due to excessive bleeding, or have you ever had to return to the operating room for hemorrhage management?
• Have you ever encountered unusually heavy menstrual periods?
• Do you notice frequent or excessive bruising? If so, are these bruises widespread, or are they primarily localized to specific areas prone to trauma?
• Have you experienced recurrent nosebleeds, either currently or at any point in your life?
• Have you ever undergone a blood or plasma transfusion? If yes, could you provide details regarding the circumstances that necessitated this procedure?
• Have you ever sustained bruising or bleeding as a result of trauma, such as car accidents, falls, sports injuries, altercations, or other violent incidents?

These contents were adapted from Consultative Hemostasis and Thrombosis (Fourth Edition) [14]

**Table 2:** Schema for preoperative hemostatic evaluation

**Level of Risk**^**a**^	**Screening History**	**Proposed Surgery**	**Recommended Tests**
**Minimal**	Negative ± prior surgery *and*	Minor	None
**Low**	Negative with prior surgery* and*	Major	Platelet count, PTT, or none
**Moderate**	Possible bleeding disorder *or*	CNS, CPB, or prostatectomy	Above tests plus BT (or PFA),^b^ PT
**High**	Highly suspicious or documented bleeding disorder *and*	Major or minor	Above tests plus factors VIII, IX, and XI levels, TT. If these are negative, pursue diagnosis

These contents were adapted from Consultative Hemostasis and Thrombosis (Fourth Edition) [14].

*Abbreviation: BT* bleeding time, *CNS* central nervous system, *CPB* cardiopulmonary bypass, *PFA* platelet function assay, *PTT* partial thromboplastin time, *PT* prothrombin time, *TT* thrombin time

^a^Estimated by the product of the risk of bleeding times the clinical consequence of bleeding.

^b^The bleeding time may be replaced by PFA

### Preoperative PLT transfusion

The human hemostatic system maintains a delicate balance between procoagulant and anticoagulant mechanisms. Procoagulant actions include PLT adhesion, aggregation, and fibrin clot formation, while anticoagulant mechanisms involve the natural inhibitors of coagulation and fibrinolysis. Typically, hemostasis is finely regulated to facilitate blood flow while also being prepared for rapid clot formation to stop bleeding and prevent excessive blood loss. The key components of this system include (1) PLTs and other blood components, such as monocytes and erythrocytes; (2) plasma proteins, including coagulation and fibrinolytic factors and inhibitors; and (3) vessel walls [15]. Preoperative PLT transfusion is employed in clinical practice to reduce the risk of bleeding during and after surgery; however, PLTs are only one component of the hemostatic process.

The relationship between a decrease in PLT count and the risk of nontraumatic bleeding remains undefined, with several clinical factors needing consideration. The PLADO study, a multicenter, randomized, controlled trial, evaluated the association between PLT count, transfusion practices, and bleeding outcomes in patients undergoing chemotherapy or stem cell transplantation (SCT) at risk for developing hypoproliferative thrombocytopenia [16]. The study enrolled 1,077 patients, including 378 who underwent autologous SCT, 413 who underwent allogeneic SCT, and 286 who received chemotherapy for hematologic malignancies. Patients with a PLT count of ≤5 × 10^9^/L demonstrated a higher bleeding risk compared with those with a PLT count of ≥81 × 10^9^/L. In the multivariate analysis of grade 2 or greater bleeding, laboratory parameters associated with increased bleeding included a PLT count of ≤5 × 10^9^/L, a hematocrit count of ≤25%, an INR of >1.2, and an aPTT of >30 s. However, in a multivariate analysis of grade 3 or higher bleeding requiring red blood cell (RBC) transfusion, PLT count was not significantly associated. Additionally, PLT transfusions administered on the days of bleeding often prove insufficient to alter bleeding outcomes the following day. In another study of hospitalized patients undergoing SCT or chemotherapy for hematologic cancers or solid tumors, where prophylactic PLT transfusions were administered based on thresholds ranging from approximately 20–70 × 10^9^/L, prophylactic PLT transfusion did not affect the incidence of bleeding [17].

Studies on the optimal PLT threshold and appropriate transfusion dose to reduce the bleeding risk remain limited. The PLT thresholds recommended for different procedures according to each guideline are summarized in Table 3 [18–22].

**Table 3:** Transfusion guideline recommendations for prophylactic PLT transfusions

**Society/recommendations**	**Strength of recommendation/** **Quality of evidence**
**European Society of Intensive Care Medicine 2020 **[17]	
We suggest refraining from platelet transfusions to treat thrombocytopenia unless the platelet count falls below 10 × 10^9/L.	Conditional/ Very low
We make no recommendation regarding prophylactic platelet transfusion before invasive procedures for platelet counts between 10 × 10^9/L and 50 × 10^9/L.	Research recommendation/ -
We suggest refraining from prophylactic platelet transfusion before percutaneous tracheostomy or central venous catheter insertion for platelet counts between 50 × 10^9/L and 100 × 10^9/L.	Conditional/ Very low
**Society of Interventional Radiology 2019 **[18]	
Consider platelet transfusion if the platelet count is <20 × 10^9/L for procedures with low bleeding risk (e.g., central venous access, including PICC placement, dialysis access, lumbar puncture, paracentesis, thoracentesis, transjugular liver biopsy, or superficial abscess drainage).	Weak/ Limited(evidence level D)
Consider platelet transfusion if the platelet count is <50 × 10^9/L for procedures with high bleeding risk (e.g., deep abscess drainage, solid organ biopsies, arterial intervention <7 French sheath, gastrostomy, urinary tract interventions [nephrostomy, stone removal], or transjugular intrahepatic portosystemic shunt).	Weak/ Limited(evidence level D)
**British Society for Haematology 2017 **[19]	
Consider performing the following procedures above the platelet count threshold indicated: Central venous lines, >20 × 10^9/L (using ultrasound).	Strong/ Moderate
Major surgery, >50 × 10^9/L.	Strong/ Low
Lumbar puncture, ≥40 × 10^9/L.	Weak/ Low
Insertion/removal of an epidural catheter, ≥80 × 10^9/L.	Weak/ Low
Neurosurgery or posterior segment ophthalmic surgery, >100 × 10^9/L.	Strong/ Low
Percutaneous liver biopsy, >50 × 10^9/L (consider transjugular biopsy if platelet count is lower).	Weak/ Moderate
Provide prophylactic platelet transfusions (platelet transfusions to patients who do not have clinically significant bleeding and do not require a procedure) to patients with reversible bone marrow failure (e.g., general critical illness, receiving intensive chemotherapy, or undergoing hematopoietic stem cell transplantation) at or above 10 × 10^9/L.	Strong/ Moderate
Consider increasing the threshold for prophylactic platelet transfusion to between 10 × 10^9/L and 20 × 10^9/L in patients judged to have additional risk factors for bleeding (e.g., sepsis).	Weak/ Low
**American Association of Blood Banks (AABB) 2015 **[20]	
Suggest prophylactic platelet transfusion for patients having elective central venous catheter placement with a platelet count <20 × 10^9/L.	Weak/ Low
Suggest prophylactic platelet transfusion for patients having elective diagnostic lumbar puncture with a platelet count <50 × 10^9/L.	Weak/ Very low
Suggest prophylactic platelet transfusion for patients having elective neuraxial anesthesia with a platelet count <50 × 10^9/L.	Weak/ Very low
Recommends against routine prophylactic platelet transfusion for patients who are nonthrombocytopenic and have cardiac surgery with cardiopulmonary bypass.	Weak/ Very low
Recommends transfusing hospitalized patients with a platelet count <10 × 10^9/L to reduce the risk of spontaneous bleeding.	Strong/ Moderate

These contents were adapted from "How I use platelet transfusions [22]

### Preoperative RBC transfusion

In a cohort study involving adult patients aged 18 years or older (*n* = 232,440) requiring inpatient care, approximately 20% presented with anemia upon admission, while more than 50% developed anemia during admission [23]. Patients who presented anemia upon admission had significantly higher mortality rates than in those without anemia, with the risk increasing significantly if the anemia worsened during the hospitalization period. In 18 large observational studies involving more than 650,000 surgical patients, the mean prevalence of preoperative anemia was approximately 35%, ranging from 10.5% to 47.9% depending on the type of surgery [24]. After adjusting for several potential confounders, including age, sex, and underlying medical conditions, preoperative anemia emerged as an independent risk factor for increased postoperative morbidity and mortality and prolonged hospital stays [24].

The effectiveness of RBC transfusions in reducing mortality among hospitalized patients or those undergoing surgery with anemia remains uncertain. Although RBC transfusions rapidly increases hemoglobin (Hb) levels, the effectiveness of transfusions in improving tissue oxygen consumption or reducing tissue oxygen debt is still unknown. Additionally, RBC transfusions are frequently linked with poor outcomes. In critically ill patients and those undergoing surgery, transfusion of a single unit of packed RBCs is associated with a higher multivariate risk of mortality, wound complications, pulmonary problems, postoperative renal dysfunction, systemic sepsis, composite morbidity, and prolonged postoperative hospital stays compared with propensity-matched patients who did not receive intraoperative RBC transfusion [24–28]. In summary, although the postoperative mortality rate increases in patients with anemia, RBC transfusion does not decrease this rate. However, the adverse effects associated with transfusion may increase the incidence of postoperative complications and mortality.

**Fig. 1:**
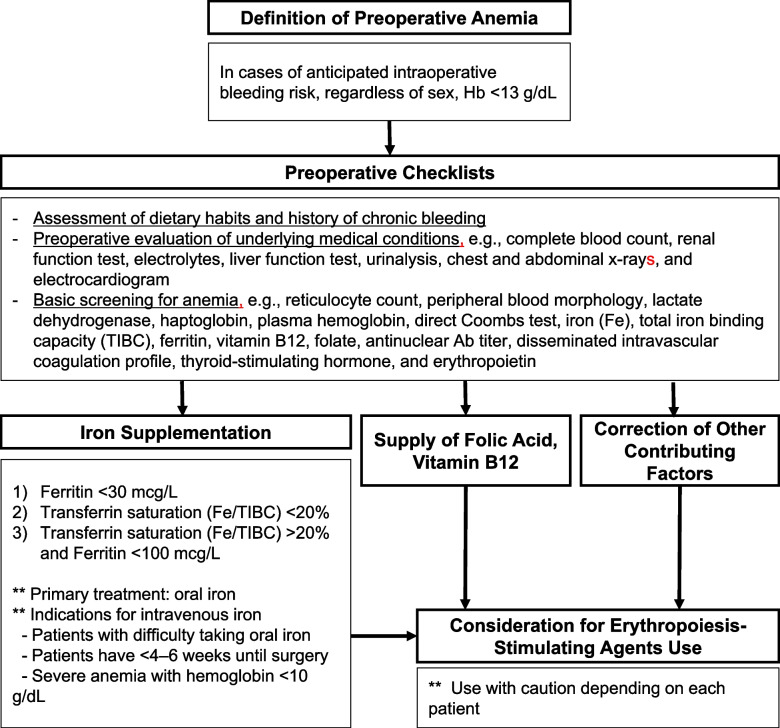
Screening and related work-up for preoperative anemia

Given the clinical drawbacks associated with both preoperative anemia and perioperative RBC transfusion, individualized strategies must be developed to minimize RBC transfusion, with the ultimate goal of improving patient outcomes. To achieve this goal, a structured approach that includes 1) the correction of preoperative anemia, 2) implementation of surgical techniques and perioperative strategies to minimize transfusion requirements, and 3) postoperative patient care is required. In alignment with the focus of this review, particular emphasis was placed on the correction of preoperative anemia. Based on the World Health Organization criteria, anemia is defined as Hb levels of <13 g/dL for adult men and <12 g/dL for adult women. However, for surgeries with the potential for blood loss exceeding 500 mL or a likelihood of transfusion of ≥10%, preoperative anemia is defined as an Hb level of <13 g/dL regardless of sex [29]. The basic checklist and the management of patients with preoperative anemia are summarized in Fig. 1.

**Fig. 2:**
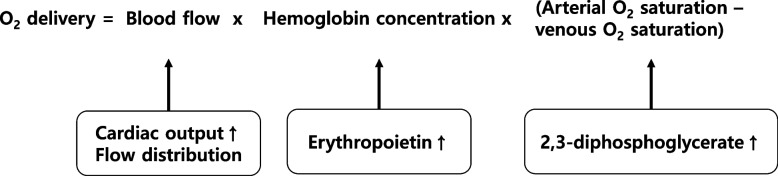
The Fick equation applied to assess anemia

The goal of correcting preoperative anemia is to stabilize oxygen delivery during surgery. The use of the Fick equation to assess anemia revealed that increasing blood flow could effectively increase oxygen delivery (Fig. 2). Additionally, erythropoiesis-stimulating agents (ESAs) are effective in increasing Hb concentration and improving oxygen delivery. The role of ESAs in managing preoperative anemia remains uncertain, with varying guidelines [30–32]. Concerns arise regarding the cardiovascular risks associated with their use in managing chronic kidney disease and cancer [33]. However, recent systematic reviews have suggested that preoperative ESA use reduces transfusion risk without increasing the incidence of thrombosis [34, 35]. When combined with iron therapy, ESAs further reduced the need for transfusions without significantly increasing the incidence of adverse events. Short-term ESA use (2–4 weeks) appears safe in the preoperative setting; however, its cost-effectiveness and overall impact on patient outcomes warrant further investigation.

## Conclusion

Identifying and correcting remediable or modifiable risk factors before surgery is critical for reducing postoperative complications and mortality. Most cases of postoperative bleeding stem from surgical factors rather than hemostatic failure, and conventional preoperative screening tests may not reliably predict surgical bleeding. The assessment of bleeding risk through history taking and physical examination, followed by selective preoperative screening tests in high-risk patients, is recommended. The effectiveness of preoperative PLT transfusion in managing bleeding risk remains inconclusive. Similarly, the transfusion of RBCs in patients with anemia does not reduce the risk of postoperative mortality and may increase the incidence of complications. Individualized strategies focused on correcting preoperative thrombocytopenia or anemia and minimizing transfusion requirements through surgical techniques and perioperative strategies are essential for improving patient outcomes [36].

## Data Availability

No datasets were generated or analysed during the current study.
